# Evaluation of the Therapeutic Effect of Curcumin-Conjugated Zinc Oxide Nanoparticles on Reserpine-Induced Depression in Wistar Rats

**DOI:** 10.1007/s12011-023-03849-z

**Published:** 2023-09-15

**Authors:** Heba M. Fahmy, Fatmaalzahraa A. Aboalasaad, Ayman S. Mohamed, Fathi A. Elhusseiny, Yasser A. Khadrawy, Ahmed Elmekawy

**Affiliations:** 1 Biophysics Department, Faculty of Science, Cairo University, Cairo, Egypt; 2 Physics Department, Faculty of Science, Tanta University, Tanta, Egypt; 3 Zoology Department, Faculty of Science, Cairo University, Cairo, Egypt; 4 Medical Physiology Department, Medical Division, National Research Centre, Cairo, Egypt

**Keywords:** Zinc oxide nanoparticles, Curcumin, Monoamines, Reserpine, Depression

## Abstract

Depression, a devastating brain illness, necessitates the exploration of novel antidepressant treatments. We evaluated the antidepressant effects of free curcumin, zinc oxide nanoparticles (ZnO NPs), and curcumin-conjugated zinc oxide nanoparticles (Zn(cur)O NPs). The nanoformulations were extensively characterized using advanced techniques. An acute toxicity study ensured the safety of Zn(cur)O NPs. Rats were assigned to one of five groups: control, reserpine-induced depression model, treatment with ZnO NPs, free curcumin, or Zn(cur)O NPs. Behavioral assessments (forced swimming test [FST] and open-field test [OFT]) and neurochemical analyses were conducted. Zn(cur)O NPs exhibited superior efficacy in ameliorating reserpine-induced behavioral and neurochemical effects compared to free curcumin and ZnO NPs. The reserpine-induced model displayed reduced motor activity, swimming time, and increased immobility time in the FST and OFT. Treatment with Zn(cur)O NPs 45 mg/kg significantly improved motor activity and reduced immobility time. Furthermore, Zn(cur)O NPs decreased malondialdehyde (MDA) levels while increasing reduced glutathione (GSH) and catalase (CAT) levels. Additionally, concentrations of serotonin (5-HT) and norepinephrine (NE) increased. In conclusion, curcumin-conjugated zinc oxide nanoparticles demonstrate potent antidepressant effects, alleviating depressive-like behavior in rats. These findings support Zn(cur)O NPs as a promising therapeutic strategy for depression management, warranting further investigation and clinical validation.

**Supplementary Information:**

The online version contains supplementary material available at 10.1007/s12011-023-03849-z.

## Introduction

Depression, a neuropsychiatric disease, is characterized by subtle cellular and molecular changes that affect a network of neural substrates [1]. Its manifestations encompass persistent sleep disturbances, apathy, anhedonia, reduced social interest, anorexia, and malaise [2, 3]. Depressive individuals face increased morbidity and mortality risks for various reasons [4]. The pathophysiology of depression has been elucidated through multiple theories, including the monoaminergic hypothesis involving serotonin and noradrenaline [5]. Furthermore, peripheral immune system activation and neuroinflammation have also been implicated as potential contributors to depression [6]. Curcumin, a hydrophobic polyphenol derived from the rhizome of the *Curcuma longa* herb, exhibits diverse biological and pharmacological activities [7]. As the most active component of turmeric, a popular Indian spice, curcumin typically constitutes 2–8% of turmeric formulations [8]. This compound demonstrates potent antioxidant properties, effectively suppressing oxidative stress markers [9, 10]. Furthermore, curcumin modulates immune inflammation by inhibiting COX-2, reducing levels of pro-inflammatory cytokines [11, 12]. Its neuroprotective effects have been extensively observed [13], significantly benefiting neuronal health [14, 15]. Additionally, curcumin influences the activity of the hypothalamic–pituitary–adrenal (HPA) axis, a key regulatory system involved in stress response [14, 16]. Notably, curcumin affects the transmission of monoamine neurotransmitters, exerting effects on serotonergic and dopaminergic activities [16–18].

Nanoparticles hold promise for many diseases, benefiting from their unique characteristics such as high lipid solubility, ability to cross the blood–brain barrier, and enhanced reactivity [19]. They find applications in various domains of medicine and biology [20]. Within the nanoscale range of 1 to 100 nm, nanoparticles can traverse biological barriers, including the blood–brain barrier (BBB). Leveraging nanoparticles as drug delivery systems presents a strategy to facilitate drug molecules’ passage across the BBB [21]. Such drug delivery systems offer numerous advantages, including targeted delivery to specific organs or tissues, improved bioavailability, safer formulations, sustained release of medications, and extended circulation time in the bloodstream [22]. Zinc (Zn) plays a critical role in living organisms, influencing the activity of over 300 enzymes involved in DNA proliferation, transcription, and protein synthesis.

Furthermore, Zn is essential for cellular division and differentiation [23–25]. The brain exhibits the highest concentration of Zn, particularly in the hippocampus and cerebral cortex [26]. Previous studies have demonstrated that zinc oxide nanoparticles (ZnONPs) administered at doses of 5 or 10 mg/kg enhance locomotor activity, exhibit anxiolytic effects, and improve spatial memory and cognitive deficits in rats [27]. Based on the previous studies, zinc oxide nanoparticles and curcumin have positively impacted depression. Therefore, the notion arose that by combining them in a formulation, a notably effective depression treatment could be achieved.

In this study, we investigated the antidepressant-like activity of curcumin-conjugated zinc oxide nanoparticles (ZnO NPs) in Wistar adult male rats with reserpine-induced depression. Additionally, we aimed to explore the potential underlying mechanisms associated with this activity.

## Materials and Methods

### Materials

Zinc nitrate hexahydrate (Zn(NO_3_)_2_·6H_2_O) (98% extra pure), potassium hydroxide, and acetone (HPLC grade) were obtained from the International Trade Company. The curcumin and reserpine used in this study were provided by Sigma-Aldrich.

### Methods of Preparation of Zinc Oxide Nanoparticles (ZnO NPs) and Curcumin-Conjugated Zinc Oxide Nanoparticles (Zn (Cur)O NPs)

Zinc oxide nanoparticles (ZnO NPs) and curcumin-conjugated zinc oxide nanoparticles (Zn(cur)O NPs) were synthesized using the method outlined by Moussawi et al. [28]. It is worth mentioning that after preparing the ZnO NPs, the sample was characterized using techniques like Fourier-transform infrared (FTIR) and UV–vis. spectroscopy, and this information is provided in the supplementary material. To prepare Zn(cur)O NPs, 2 mg of curcumin was dissolved in 50 mL of double-distilled water at temperatures between 80 and 90 °C while stirring magnetically. Once the curcumin was fully dissolved, a solution containing 50 mL of 0.1 M zinc nitrate (Zn(NO_3_)_2_·6H_2_O) in double-distilled water was added. The resulting solution was amber and refluxed at temperatures ranging from 85 to 90 °C for 1 h. After cooling, the solvent was removed, and the remaining solution was adjusted to a volume of 5 mL using 0.2 M potassium hydroxide (KOH) and stored at 4 °C, resulting in a pale yellowish liquid. The solution was then centrifuged at 5000 rpm, and the precipitate was washed with water until the yellow color disappeared. Then, acetone washing was conducted to extract any unattached curcumin, and a final wash with water was performed. The resulting Zn(cur)O precipitate was dehydrated at room temperature under vacuum conditions.

For the synthesis of ZnO NPs, 50 mL of 0.1 M zinc nitrate (Zn(NO_3_)_2_·6H_2_O) solution was slowly added to distilled water, and 5 mL of 0.2 M KOH solution was added gradually with continuous stirring in an ice bath set to 4 °C. The resulting white suspension was then centrifuged at 5000 rpm for 5 min.

### Physical and Chemical Characterization of the Prepared Formulations

#### Transmission Electron Microscopy (TEM)

The crystalline and morphological composition of nanoparticles were examined using a TEM (JEM 1230 electron microscope Jeol, Tokyo, Japan).

#### Scanning Electron Microscope (SEM)

The surface morphology for the ZnO NPs and Zn(cur)O NPs was determined by a scanning electron microscope (Quanta™250 FEG, FEI; USA) equipped with an energy-dispersive X-ray spectrometer. The accelerating voltage was 20 kV**.**

#### Particle Size and Zeta Potential

The particle size and zeta potential of the two produced formulations (ZnO – Zn(cur)O) were determined at 25 °C using dynamic light scattering (DLS) analysis (Zeta sizer Nano ZS90, Malvern Instruments, UK).

### *In Vivo* Experiments

#### Experimental Animals

A total of 35 male adult Wistar rats, aged 8–10 weeks and weighing between 150 and 200 g, were included in this study. The diet administered to the animals in this study, designated as the “Rat chow diet,” was sourced from El Gomhorya Company, Ismailia, Egypt. This diet has an energy content of 3.06 kcal/g, comprising 21% protein, 48.8% carbohydrates, and 3% fat. Additionally, it contains 0.8% calcium, 0.4% phosphorus, 5% fiber, 13% moisture, and 8% ash. The rats were allocated into groups of 7 animals per group, resulting in 5 groups. The animals were housed under controlled conditions, maintaining a temperature of 20 ± 3 °C, a humidity level of 50 ± 3%, and a 12-h light–dark cycle. Throughout the experiment, the rats had free access to food and water. A 2-week acclimatization period was provided before the commencement of the experimental procedures. The Institutional Animal Ethics Committee reviewed and approved the protocol for the in vivo study, with registration number CU I F 51 19.

#### Acute Toxicity Study (LD50)

The LD50 (lethal dose 50%) of Zn(cur)O NPs was determined by administering different doses orally, as described by Chinedu et al. [29]. Male Wistar rats were subjected to an overnight fasting period and then divided into groups with two rats per group. Various doses (10, 100, 300, and 600 mg/kg) of the Zn(cur)O NPs solution were orally administered to the respective groups. The animals were closely observed for 1 h after administration, followed by 10-min intervals every 2 h for 24 h. Changes in behavior such as paw licking, fatigue, semi-solid stool, salivation, writhing, and loss of appetite were monitored in addition to mortality. The LD50 value was calculated using the formula LD50 = (M0 + M1)/2 = (300 + 600)/2 = 450 mg/kg, where M0 represents the highest non-mortality dose of Zn(cur)O NPs and M1 represents the lowest dose causing mortality.

#### Experimental Design

Animals were classified into five groups:

The rats were divided into different groups based on the treatment they received. Group 1, the negative control group, was injected intraperitoneally (IP) with saline. Group 2, the positive control group, received IP injections of 0.2 mg/kg reserpine daily for 14 days. Group 3 was subjected to IP injections of 0.2 mg/kg reserpine daily for 14 consecutive days, followed by oral administration of free curcumin at a dose of 45 mg/kg for 10 successive days. Group 4 received IP injections of 0.2 mg/kg reserpine daily for 14 consecutive days, followed by oral administration of ZnO NPs at a dose of 5.6 mg/kg for 10 successive days. Group 5 underwent IP injections of 0.2 mg/kg reserpine daily for 14 consecutive days, followed by oral administration of Zn(cur)O NP treatment at a 45 mg/kg dose for 10 successive days. Reserpine was administered via IP injections, while the drugs (curcumin, ZnO NPs, and Zn(cur)O NPs) were administered orally. The evaluation of rat behaviors before and after treatments was performed through the following behavioral tests:

### Behavioral Tests

#### The Open-Field Test (OFT)

**Fig. 1:**
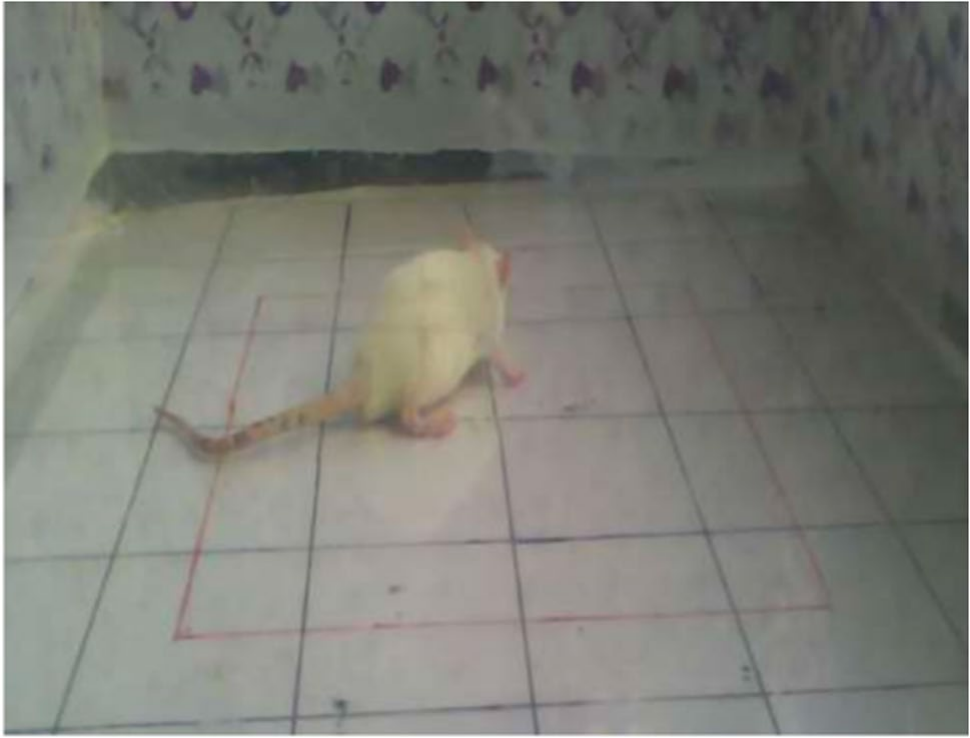
Open-field test

The open-field test (OFT) was employed in this study to evaluate the behavioral changes in the rats. The test was conducted twice, first on the 15th day after inducing the rat model of depression and then on the 10th day after administering the treatment. The OFT is a widely used method to assess various parameters related to the rats’ activity levels, locomotor activity, and exploratory behavior [29]. This test provides valuable insights into the treatments’ impact on the rats’ overall behavior, allowing for a comprehensive evaluation of their response to the experimental conditions. The grooming process starts as the mouse licks its paws. Subsequently, the mouse employs its damp paws to gently brush the nose in elliptical motions. Once the nose is clean, it utilizes one paw at a time to groom the whiskers and the vicinity of the eyes. Finally, it employs both paws to groom the head with upward and backward strokes. The healthy rats serve as the negative control, and the number of squares must be greater than the model group injected with reserpine. The OFT test is shown in Fig. 1.

#### The Forced Swimming Test (FST)

**Fig. 2:**
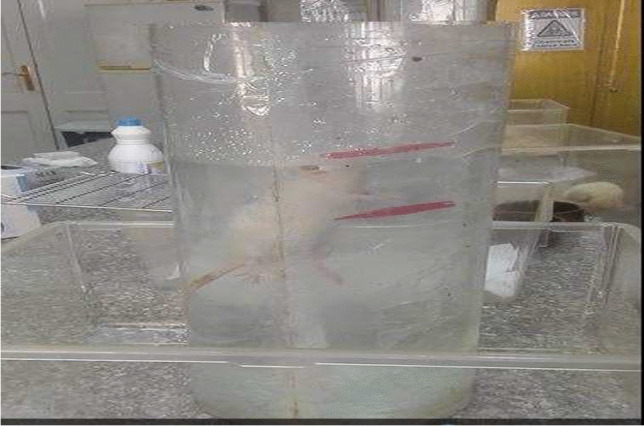
Forced swimming test

On the 15th and 16th days after inducing the depression model and on the 10th day post-treatment, FST was administered. Each rat was individually placed in a spacious Plexiglas tank filled with water, occupying around 75% of the tank’s capacity to prevent it from reaching the bottom. The tank had a cylindrical tube measuring 50 cm in height and 22.5 cm in diameter. The FST was conducted under standard conditions at room temperature, following the methodology outlined by Kumar et al. [30]. The FST test is shown in Fig. 2.

The swimming behavior of the rats in both the OFT and FST was documented using specialized software. Afterward, the collected data underwent analysis using analysis of variance (ANOVA) followed by post hoc tests. Once the behavioral tests were concluded, the rats were euthanized, and their brains were carefully dissected, specifically separating the cortex, hippocampus, and striatum. The brain regions were weighed and then frozen at − 4 °C until further biochemical analyses were conducted at room temperature at 22 °C. Each brain region was homogenized in phosphate buffer (pH 7.4) and subsequently centrifuged at 5000 rpm. The resulting supernatant was utilized for the measurement of various endpoints:

### Determination of Lipid Peroxidation

According to Ruiz-Larrea et al. [31], malondialdehyde (MDA) is an end product of lipid peroxidation. MDA interacts with thiobarbituric acid to generate a pink complex in this test. The absorbance of the resultant complex was measured using a spectrophotometer at 532 nm (Jenway UV-6420; Barloworld Scientific, Essex, UK).

### Determination of Reduced Glutathione Level (GSH)

Ellman’s approach detected low levels of glutathione (GSH) [32]. The -SH groups of GSH reduce Ellman’s reagent during the production of 2-nitro-s-mercaptobenzoic acid. The bright yellow color of nitro mercaptobenzoic acid was determined spectrophotometrically at 412 nm.

### Determination of Catalase (CAT)

Catalase activity (CAT) was assessed following the procedure outlined by Aebi [33]. A specific quantity of hydrogen peroxide was allowed to react with CAT for 1 min, and the reaction was subsequently halted using a CAT inhibitor. In the presence of peroxidase, any remaining hydrogen peroxide reacted with 3,5-dichloro-2-hydroxybenzene sulfonic acid and 4-aminophenazone, resulting in the formation of a chromophore with color intensity inversely proportional to the activity of CAT. The absorbance of the chromophore at 510 nm was measured using a Thermo Spectronic Helios Alpha spectrometer (UVA 111615, England).

### Monoamine Measurements

Initially, the cortex, hippocampus, and striatum were homogenized using an ice-cold solution containing acidified n-butanol. Subsequently, the homogenates were centrifugated at 2000 rpm for 5 min. Afterward, a supernatant fluid of 2.5 mL was meticulously transferred into tubes containing 1.6 mL of 0.2 N acetic acid and 5 mL of heptane. Following a vortex mixing duration of 30 s and centrifugation at 2000 rpm for 5 min, the organic supernatant was discarded. From the resultant aqueous phase, a specific sample of 200 µL was extracted to precisely measure serotonin (5-HT) levels using o-phthalaldehyde. In a separate sample measuring 1 mL, the levels of norepinephrine (NE) and dopamine (DA) were determined by boiling in a water bath with iodine. The levels of DA, NE, and 5-HT in the supernatants were quantified using a fluorometric technique. Fluorescence measurements were performed using a spectrofluorometer (model Jasco-FP-6500, Japan) with a 150 W xenon arc lamp source. The slit bandwidth for both the excitation and emission monochromator was set to 5 nm [34].

**Fig. 3:**
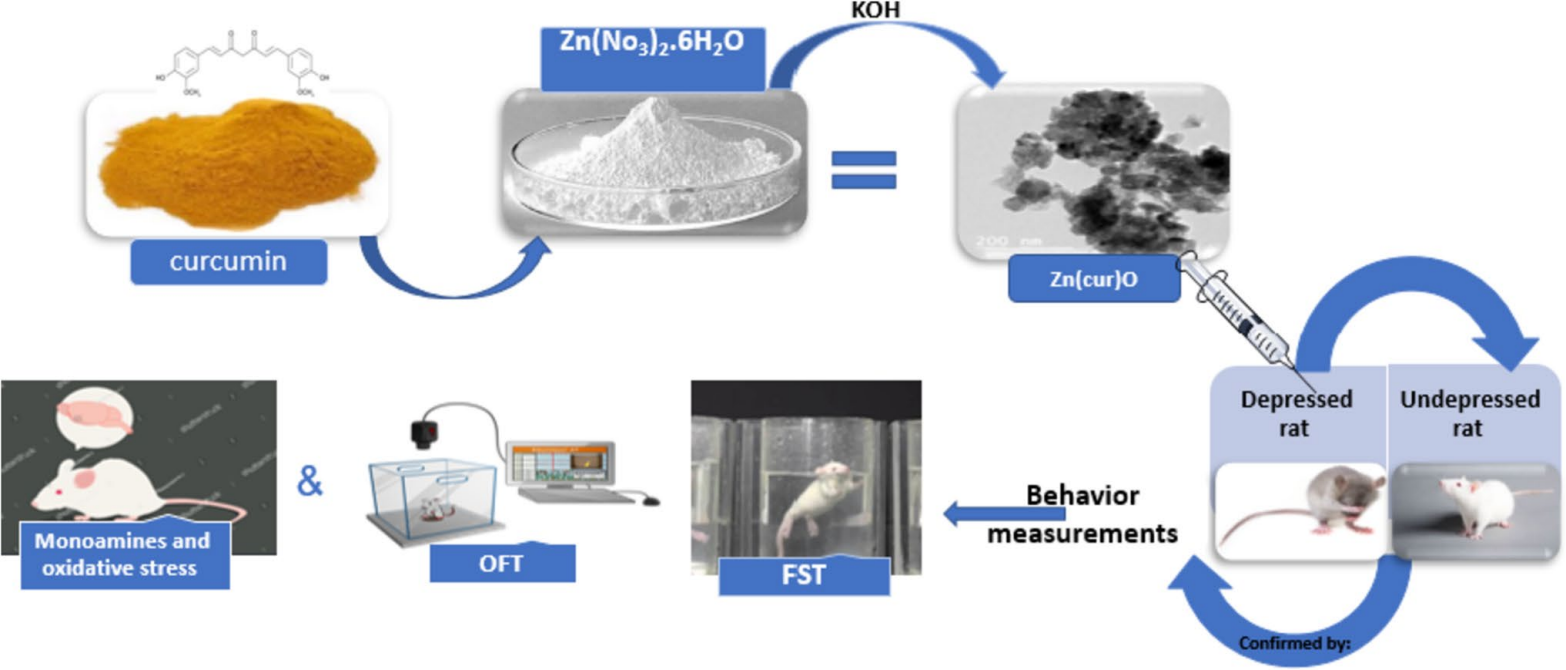
Scheme of experimental procedures for curcumin-conjugated zinc oxide nanoparticles on depressed in Wistar rats

Finally, Fig. 3 shows the scheme of experimental procedures that induces the curcumin-conjugated zinc oxide nanoparticles on depressed Wistar rats.

## Results

### The Developed Formulations’ Physical and Chemical Characteristics

#### Transmission Electron Microscope (TEM)

**Fig. 4:**
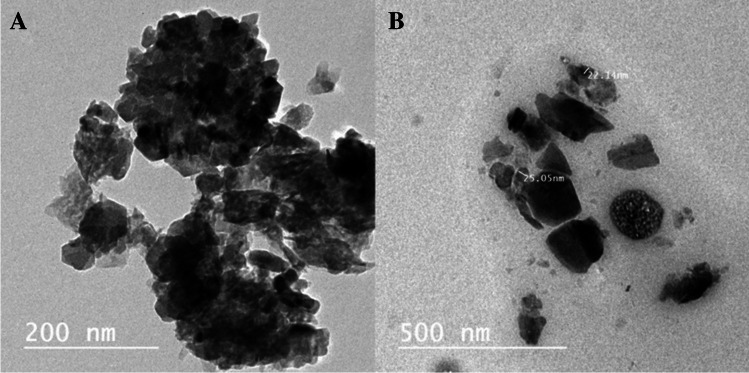
TEM micrographs of ZnO NPs with a mean diameter of 23.6 nm ± 1.46 (**A**) and Zn(cur)O with a mean diameter of 23.03 nm ± 5.2 (**B**)

Transmission electron micrographs for Zn(cur)O NPs and ZnO NPs are shown in Fig. 4A and B. TEM pictures indicated the existence of regular and somewhat homogenous particles. ZnO NPs were nearly hexagonal with a smooth surface and few aggregations, whereas Zn(cur)O NPs had a hexagonal shape.

#### Scanning Electron Microscope (SEM)

**Fig. 5:**
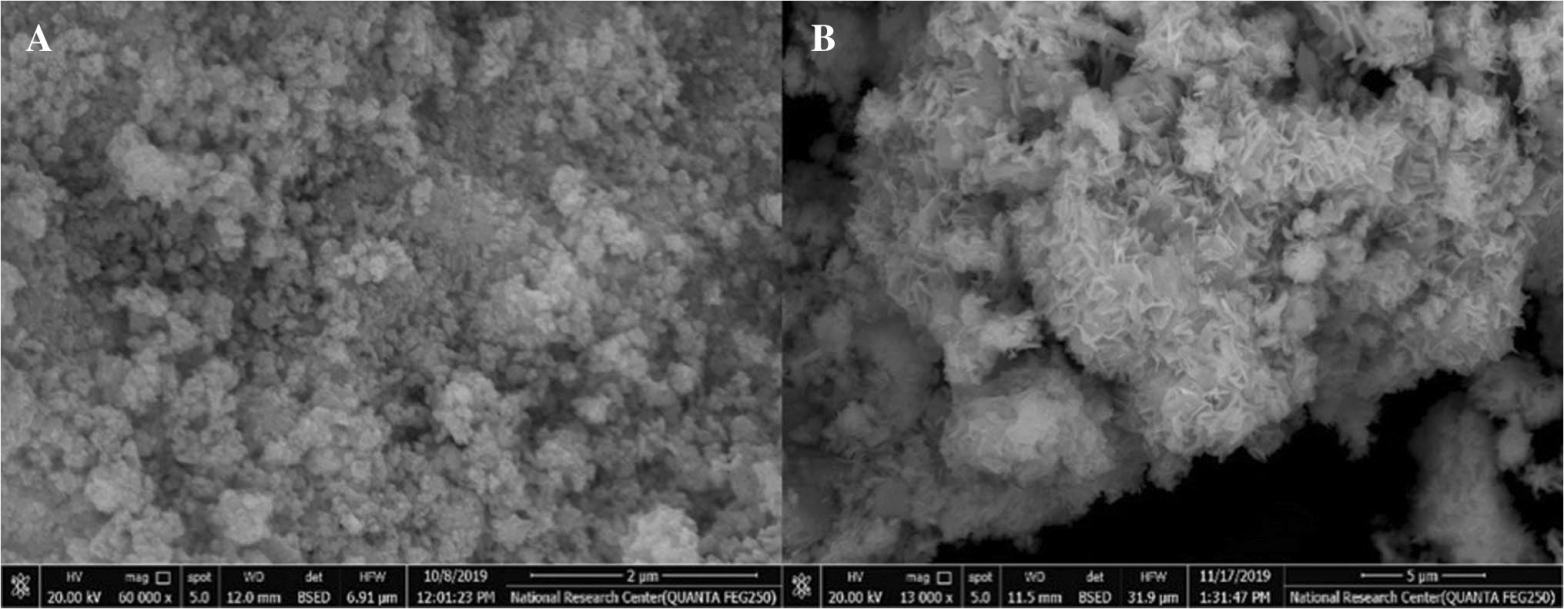
SEM of Zn(cur)O NPs (**A**) and ZnO NPs (**B**)

Scanning electron images for Zn(cur)O NPs and ZnO NPs are shown in Fig. 5A and B. Zn(cur)O NPs exhibited a spherical shape, whereas ZnO NPs had a rod shape.

#### Particle Size and Zeta Potential

**Fig. 6:**
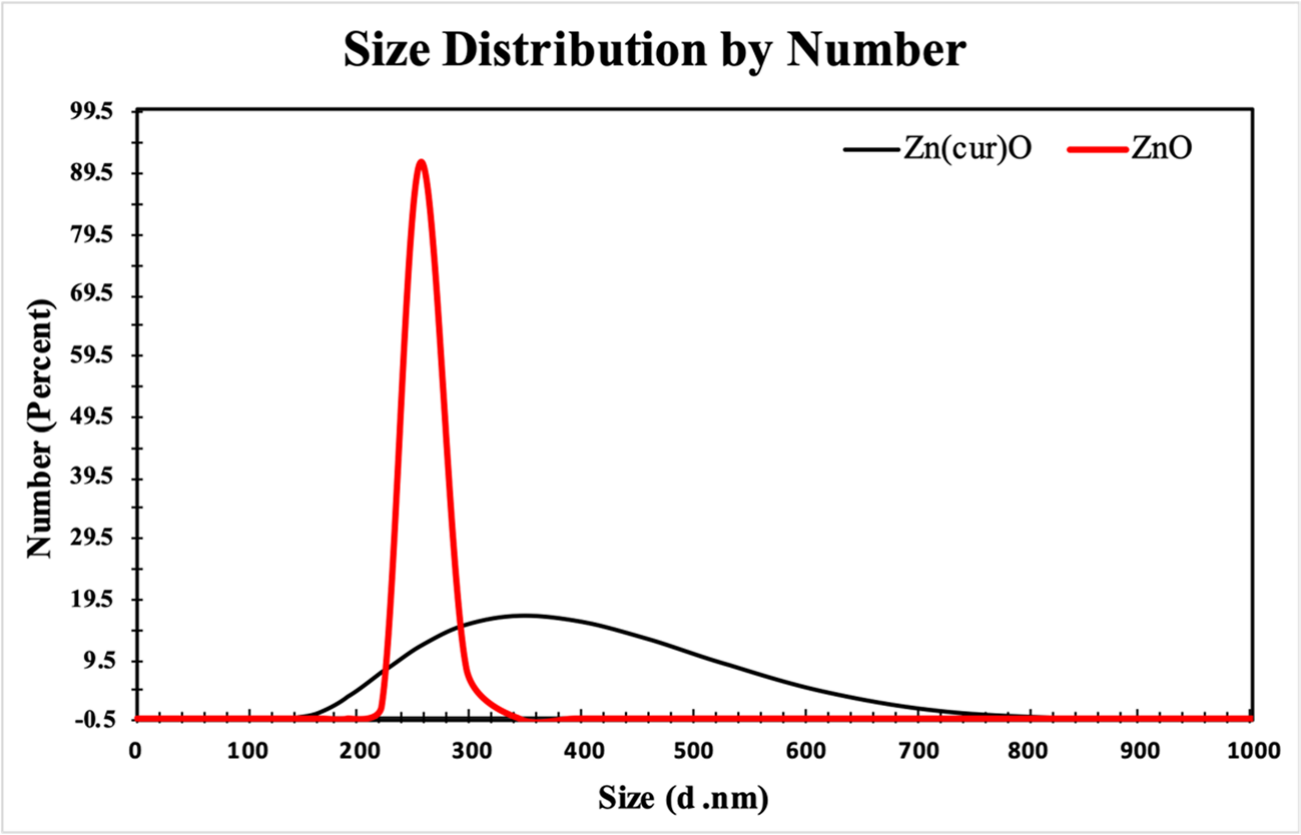
Dynamic light scattering of ZnO NPs and Zn(cur)O

The predicted hydrodynamic particle sizes for various formulations of ZnO NPs and Zn(cur)O NPs as depicted in Fig. 6 were 220 ± 11.74 nm and 342 ± 22.3 nm, respectively. For both ZnO NPs and Zn(cur)O NPs, the polydispersity index distribution (PDI) was 1.

#### Zeta Potential

**Fig. 7:**
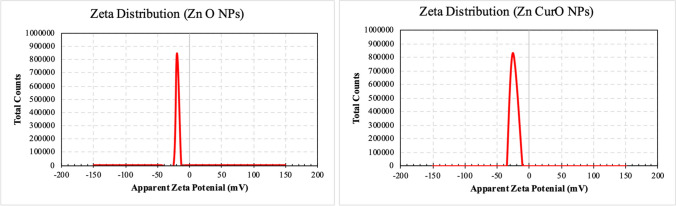
Zeta potential results of ZnO NPs and Zn(cur)O

The mean zeta potentials for ZnO NPs and Zn(cur)O NPs were − 19.6 ± 4.5 mV and − 25.6 ± 4.61 mV, respectively, as shown in Fig. 7.

### Behavioral Results

#### Open-Field Test

**Fig. 8:**
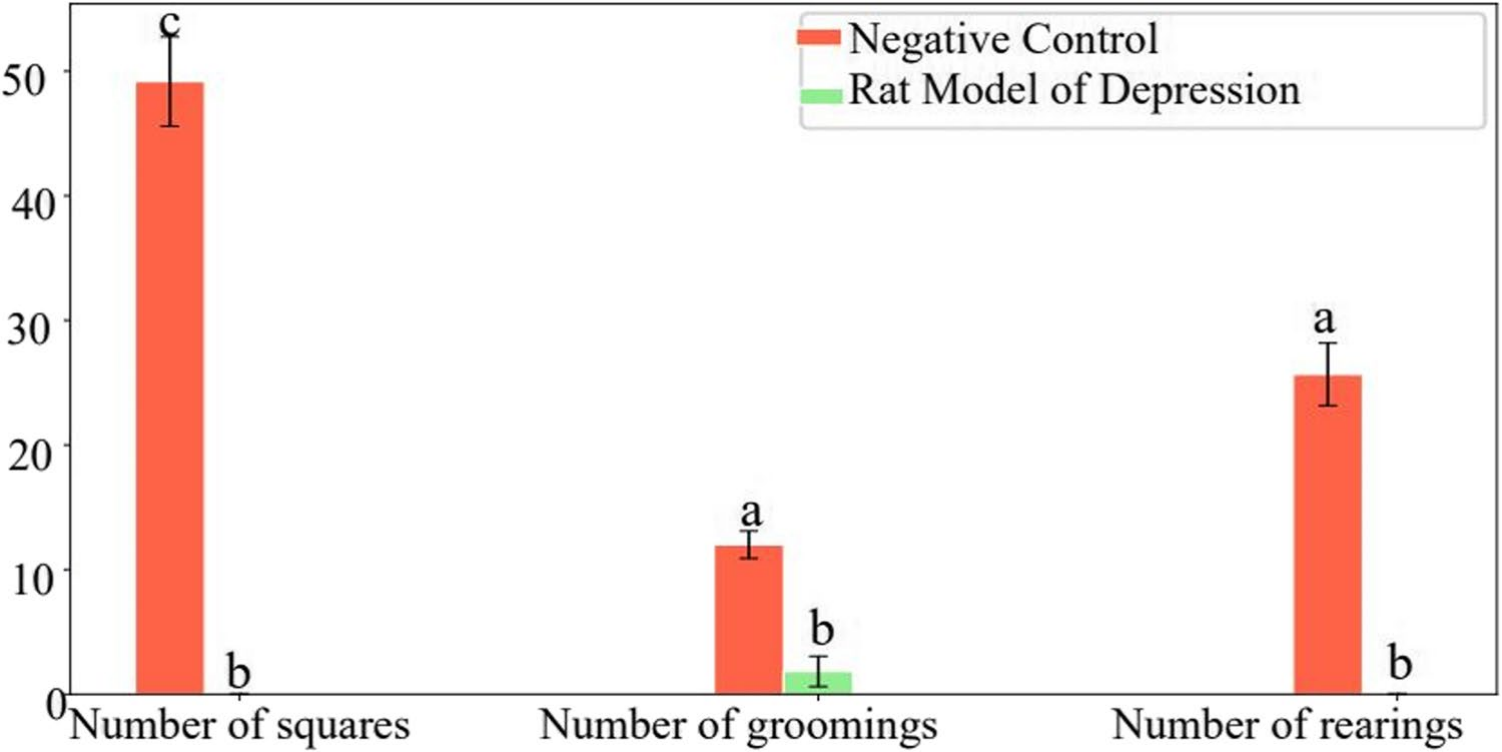
Effect of daily reserpine treatment (0.2 mg/kg) for 15 days on the open-field test (OFT). Different letters mean a significant difference between groups (at *p*-value < 0.05), and the same letters indicate non-significant changes

Using Zn(cur)O NPs as therapy resulted in insignificant changes in the grooming number compared with either the control values or the animal model of depression. The frequency of crossed squares, raising, and grooming was significantly reduced (*p* 0.05) after 15 days of daily reserpine administration which is − 100%, − 100%, and − 84.75%, respectively, below the control values (Fig. 8). After 10 days of daily treatment with different formulations, curcumin increased grooming significantly by 90.25% more than the control value. However, treatment with ZnO NPs increased grooming by 59.73%, insignificant with the animal model of depression and significant compared with control values.

**Fig. 9:**
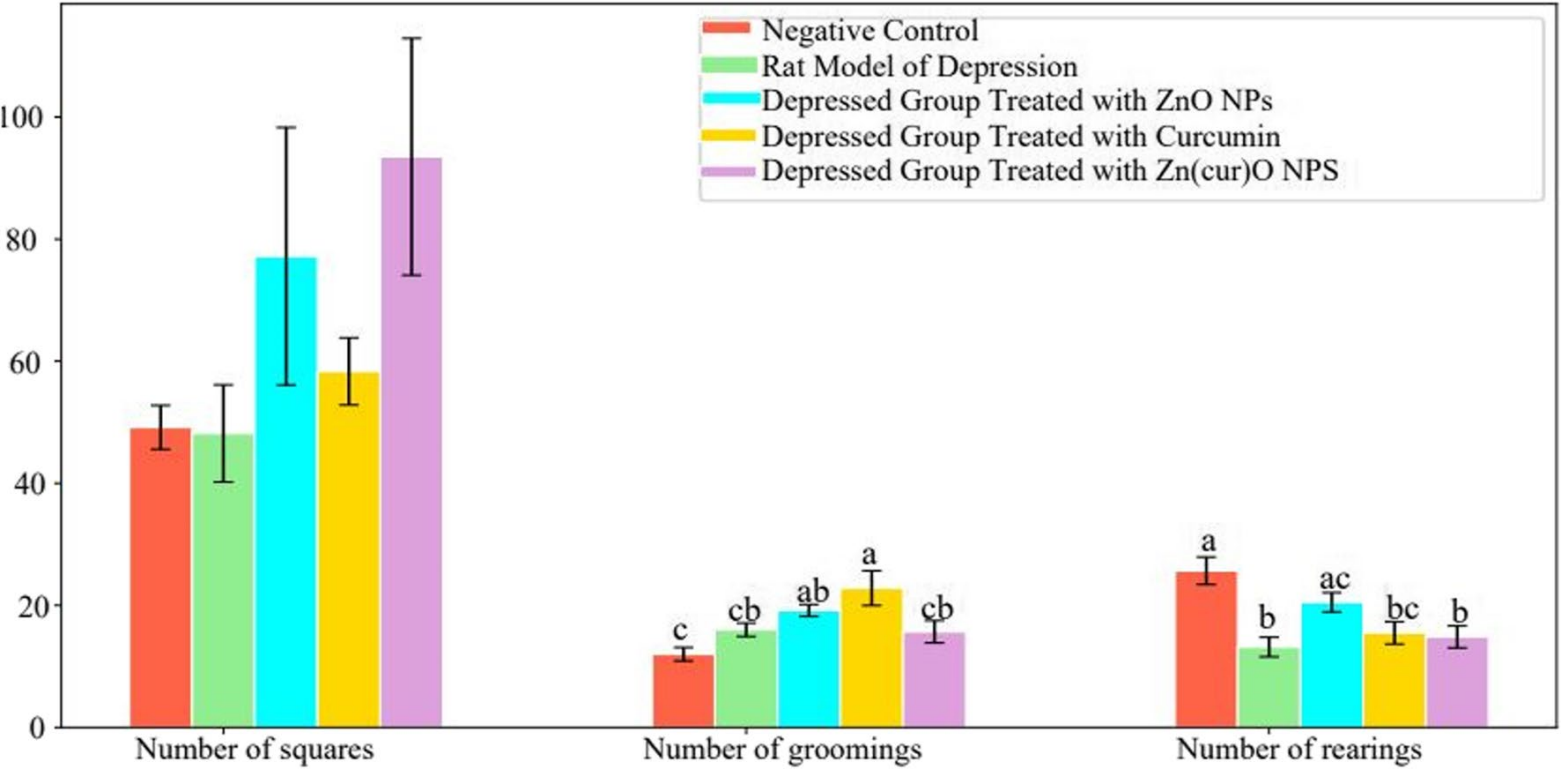
Effect of free curcumin, ZnO NPs, and Zn(cur)O on the open-field test in a rat model of depression induced by reserpine. Different letters mean a significant difference between groups (at *p*-value < 0.05), and the same letters indicate non-significant changes

After 10 days of daily treatment with ZnO NPs, a reserpine-induced rearing was reduced to − 20.14% and was significant compared with the animal model of depression and insignificant against the control group value. The use of free curcumin and Zn(cur)O NPs, on the other hand, is beneficial in insignificant changes in the rearing number compared with the animal model of depression, recording − 42.22% for the Zn(cur)O NPs and − 39.61% for curcumin against the control amount (Fig. 9).

#### Forced Swimming Test

**Fig. 10:**
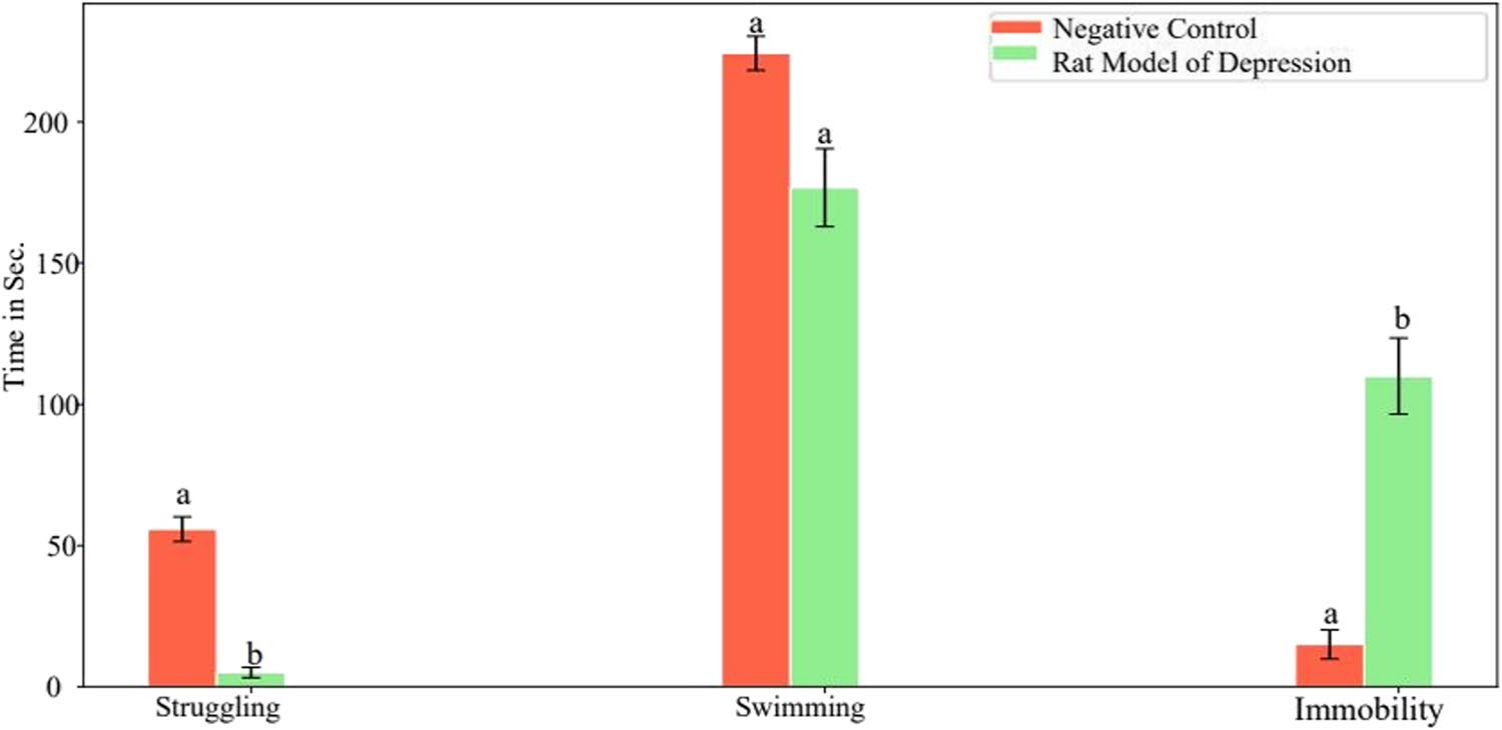
Effect of daily reserpine treatment (0.2 mg/kg) for 15 days on forced swimming test (FST). Different letters mean a significant difference between groups (at *p*-value < 0.05), and the same letters indicate non-significant changes

**Fig. 11:**
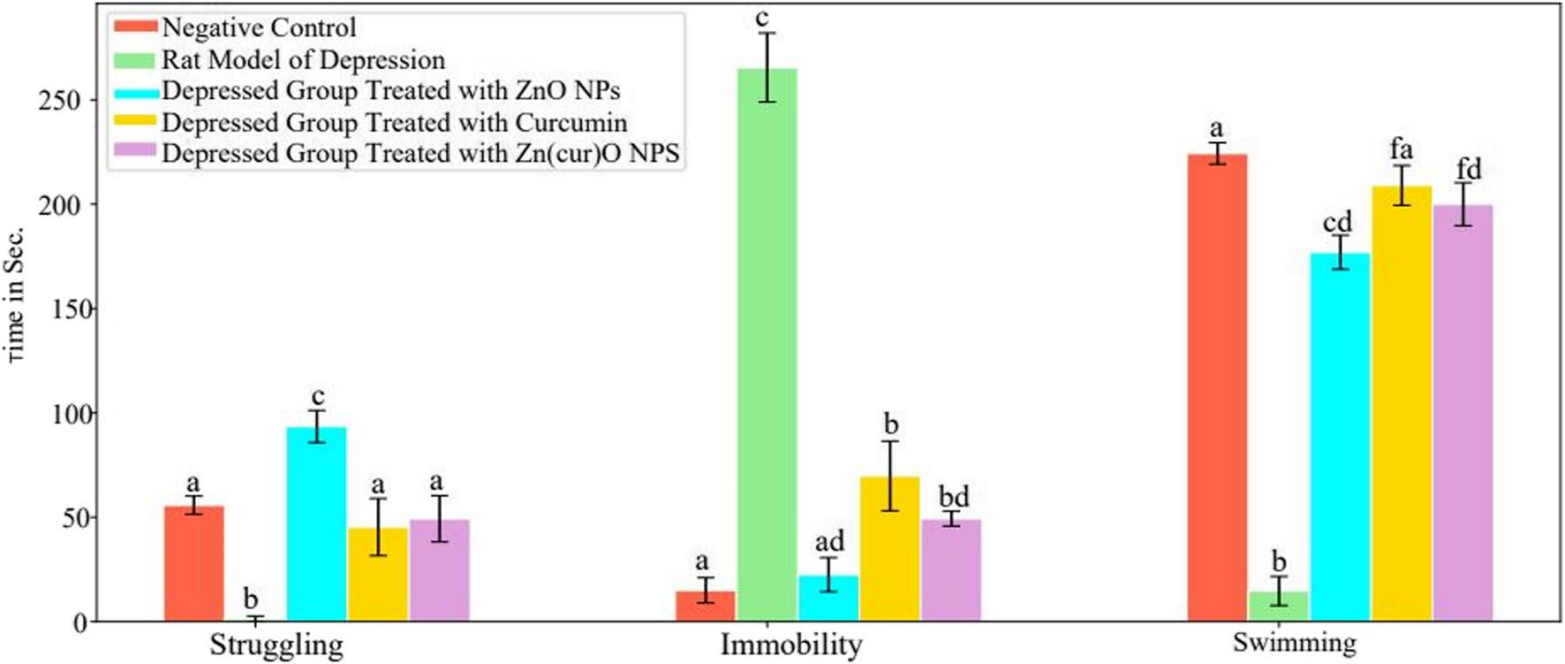
Effect of free curcumin, ZnO NPs, and Zn(cur)O on the forced swimming test in a rat model of depression induced by reserpine. Different letters mean a significant difference between groups (at *p*-value < 0.05), and the same letters indicate non-significant changes

The results indicated a statistically significant (*p* < 0.05) rise in the duration of immobility by 663.3%, a notable decrease in the time spent struggling by 91%, and a significant reduction in swimming time after 15 days of daily reserpine treatment compared to the control group values (Fig. 10). Curcumin and Zn(cur)O NP therapy decreased the increase in immobility time (1670%) generated after 15 days of daily reserpine injection to 365.3% and 228.9%. Moreover, ZnO NPs restored immobility time to an insignificant change at 50% compared with the control value. In addition, treatment with ZnO NPs and Zn(cur)O NPs increased swimming time reduction from 93.46 to 21.1% and 10.84%, respectively. However, curcumin restored the swimming time insignificantly compared with control values (6.8%). Furthermore, curcumin and Zn(cur)O NPs reduced reserpine-induced struggle time to control-like levels (Fig. 11). However, ZnO NPs significantly increased struggling time, recording 67.47% above the control values (Fig. 8).

### Neurochemical Data

#### Oxidative Stress

MDA levels in the brain rose substantially (59%), striatum (399%), and insignificantly in the hippocampus of the rat model of depression as compared to control values (Table 1).

**Table 1:** Effect of free curcumin, ZnO NPs, and Zn(cur)O NPs on the levels of MDA (nmol/g tissue) in different brain regions (hippocampus, striatum, cortex)

	Negative control	Rat model of depression	%D	Depressed rats treated with curcumin	%D	Depressed rats treated with ZnO NPs	%D	Depressed rats treated with Zn(cur)O NPs	%D
Cortex	1.44^a^ ± 0.07	2.29^bd^ ± 0.44	59	2.32^ cd^ ± 0.48	61.64	0.96^a^ ± 0.12	− 32.5	0.68^a^ ± 0.09	− 52.3
Hippocampus	4.40^a^ ± 0.74	5.83^ae^ ± 0.57	32.56	8.98^cf^ ± 0.29	104	5.25^ab^ ± 0.57	19.36	7.13^dbef^ ± 0.98	62
Striatum	4.06^a^ ± 0.34	20.26^b^ ± 3.5	399.6	10.77^c^ ± 1.60	165.6	11.51^c^ ± 1.14	183.8	10.06^c^ ± 1.23	148

Different letters mean a significant difference between groups (at p-value < 0.05) and the same letters indicate non-significant changes

There was no significant difference in the cortical MDA values with curcumin-treated rats and the depression in rat models. However, compared to the animal model of depression, there was a considerable rise in MDA levels in the hippocampus in the free curcumin group. Although free curcumin treatment reduced MDA levels significantly in the striatum compared with depressed rats, the MDA levels were still higher than the control value (Table 1).

Upon treating the rat model of depression with ZnO NPs, cortical MDA levels were restored to control-like values and decreased significantly compared with the rat model of depression. However, there was no discernible difference between the groups treated with ZnO NPs and the animal model of depression in the hippocampus. In the striatum, there was a significant reduction in MDA levels in the animal group treated with ZnO NPs comparable with the animal model of depression (Table 1).

Zn(cur)O NPs were used to treat a depressed animal model and restored cortical MDA levels to control levels, and decreased MDA levels were substantially higher than in the animal model of depression. In the striatum, Zn(cur)O NP treatment reduced MDA values significantly compared to the animal model of depression (Table 1). MDA levels were not significantly different in the hippocampus with the animal group treated with Zn(cur)O NPs and the depression model.

In the rat model of depression, an insignificant reduction in catalase activity was shown in the hippocampus (− 40.01) compared to the control group. However, a significant rise in catalase activity was shown in the hippocampus of depressed rats treated with Zn(cur)O NPs compared to all other groups investigated. The enzyme activity was not significantly changed among the studied groups (Table 2).

**Table 2:** Effect of free curcumin, ZnO NPs, and Zn(cur)O on the catalase activity (U/min/g tissue) in different brain regions (hippocampus, striatum, cortex)

	Negative control	Rat model of depression	%D	Depressed rats treated with curcumin	%D	Depressed rats treated with ZnO NPs	%D	Depressed rats treated with Zn(cur)O NPs	%D
Cortex	0.45 ± 0.03	0.48 ± 0.04	6.68	0.45 ± 0.04	0.89	0.43 ± 0.03	− 3.98	0.49 ± 0.06	9.5
Hippocampus	2.64^a^ ± 0.26	1.58^a^ ± 0.24	− 40.1	1.91^a^ ± 0.16	− 27.69	1.91^a^ ± 0.16	− 27.69	4.38^b^ ± 0.71	65.8
Striatum	1.89 ± 0.20	2.00 ± 0.20	6.3	2.46 ± 0.25	30.2	2.26 ± 0.29	19.76	2.02 ± 0.16	6.94

Different letters mean a significant difference between groups (at p-value < 0.05) and the same letters indicate non-significant changes

Curcumin treatment, compared to the control and rat models of depression, showed a considerable rise in cortical and hippocampal GSH levels and a minor change in striatal GSH levels (Table 3). A significant rise in GSH levels was observed in the striatum of the rat model of depression at 131.36% more than in the control group. However, the cortical and hippocampal GSH values did not significantly contrast with the control and the untreated groups (Table 3).

**Table 3:** Effect of free curcumin, ZnO NPs, and Zn(cur)O on the levels of GSH (nmole/g tissue) in different brain regions (hippocampus, striatum, cortex)

	Negative control	Rat model of depression	%D	Depressed rats treated with curcumin	%D	Depressed rats treated with ZnO NPs	%D	Depressed rats treated with Zn(cur)O NPs	%D
Cortex	0.41^a^ ± 0.08	0.95^a^ ± 0.16	133.11	1.57^c^ ± 0.23	284.69	1.66^c^ ± 0.27	305.7	1.84^c^ ± 0.24	349.98
Hippocampus	4.53^a^ ± 0.74	6.21^a^ ± 0.73	26.87	11.44^bd^ ± 1.48	152.31	8.62^ad^ ± 0.78	90.18	21.98^c^ ± 3.09	384.7
Striatum	1.90^a^ ± 0.13	4.40^b^ ± 0.59	131.36	4.89^b^ ± 0.42	157.36	4.49^b^ ± 0.74	136.5	6.75^c^ ± 0.78	255.37

Different letters mean a significant difference between groups (at p-value < 0.05) and the same letters indicate non-significant changes

In the cortex, there was a significant rise in GSH values in the group treated with ZnO NPs comparable with the rat model of the depression group; however, in the hippocampus and striatum, GSH levels did not differ significantly across the groups the group treated with ZnO NPs and the rat model of depression (Table 3).

In the cortex, hippocampus, and striatum, there had been a significant rise in the values of GSH in the groups treated with Zn(cur)O NPs compared with the rat model of depression at 349.98%, 384.7%, and 255.37%, respectively, more than the control values (Table 3).

#### Monoamine Neurotransmitters

Serotonin and norepinephrine levels in the depression rat model’s brain reduced scientifically at − 43.9% and − 17.91%, respectively, compared with control rats. Moreover, 5-HT, NE, and dopamine DA values exhibited significant decreases in the depressed, untreated group (− 47.2%, − 64%, and − 44.1%, respectively), comparable with the control. However, changes in monoamine neurotransmitter levels were not statistically significant in the striatum of the depressed group compared with the control group (Table 4).

**Table 4:** Effect of free curcumin, ZnO NPs, and Zn(cur)O NPs on the levels (µg/g) of serotonin (5-HT), norepinephrine (NE), and dopamine

		*Negative control*	*Rat model of depression*	*%D*	*Depressed rats treated with curcumin*	*%D*	*Depressed rats treated with ZnO NPs*	*%D*	*Depressed rats treated with Zn(cur)O NPs*	*%D*
***Cortex***	5-HT	8.72^a^ ± 0.56	4.89^b^ ± 0.58	− 43.9	4.53^b^ ± 0.402	− 48	5.37^b^ ± 0.59	− 38.4	4.80^b^ ± 0.76	− 44.95
	NE	0.79^a^ ± 0.05	0.65^b^ ± 0.04	− 17.9	0.64^b^ ± 0.04	− 19.11	0.66^b^ ± 0.03	− 16.2	1.06^c^ ± 0.06	33.86
	DA	1.81 ± 0.23	1.90 ± 0.29	4.69	2.12 ± 0.20	17.13	2.38 ± 0.36	31.32	2.38 ± 0.27	31.55
***Hippocampus***	5-HT	25.13^ac^ ± 2.3	13.26^b^ ± 2.21	− 47.2	22.22^c^ ± 1.28	− 11.57	20.91^c^ ± 2.94	− 16.79	31.42^a^ ± 2.56	25
	NE	4.38^a^ ± 0.27	1.29^b^ ± 0.10	− 64	1.84^b^ ± 0.19	− 57.9	1.66^b^ ± 0.21	− 62	1.85^b^ ± 0.45	− 57.6
	DA	19.52^a^ ± 2.03	10.91^b^ ± 0.38	− 44.1	11.49^b^ ± 0.72	− 41.13	10.75^b^ ± 0.35	− 44.9	12.47^b^ ± 2.12	− 36.11
***Striatum***	5-HT	9.85^a^ ± 0.83	6.03^a^ ± 0.23	− 38.8	9.91^a^ ± 1.9	0.59	7.99^a^ ± 0.73	− 18.9	24.25^b^ ± 3.97	146.17
	NE	1.24 ± 0.15	1.41 ± 0.12	13.3	1.07 ± 0.12	− 13.8	1.72 ± 0.31	38.7	1.69 ± 0.57	36.3
	DA	3.16 ± 0.35	3.55 ± 0.23	12.4	2.44 ± 0.54	− 22.66	4.08 ± 0.40	28.9	4.46 ± 0.78	41.3

Different letters mean a significant difference between groups (at p-value < 0.05) and the same letters indicate non-significant changes

When the depression rat model was given treatment with curcumin, there were no significant differences in the 5-HT, NE, and DA cortical values compared with the depression rat model. In the hippocampus, curcumin increased 5-HT levels significantly more than in depressed rats; however, DA and NE exhibited an insignificant change compared with the animal model of depression. There were no noteworthy changes in monoamine neurotransmitters in the striatum between curcumin-treated and depressed rats (Table 4).

There were insignificant changes in the cortical values of 5-HT, NE, and DA in the animal group treated with ZnO NPs compared with the depression rat model. In the hippocampus, there was a significant rise in 5-HT levels in the animal group treated with ZnO NPs compared with the rat model of depression. Still, there were insignificant changes in the values of DA and NE compared with the depression rat model. In the striatum, no significant changes in the levels of 5-HT, NE, or DA were observed in rats treated with ZnO NPs compared with the rat model of depression (Table 4).

Using Zn(cur)O NPs induced a significant rise in cortical values of NE; however, 5-HT and DA showed insignificant differences compared with the depression rat model. In the hippocampus, there was a significant rise in 5-HT levels and insignificant changes in NE and DA in the animal group treated with Zn(cur)O NPS compared with the rat model of depression. Zn(cur)O NPs increased 5-HT significantly by 146.17% more than the control values (Table 4).

The results were presented as mean S.E.M. (standard error of mean) and evaluated using a Student *t*-test with a significance level 0.05. The standard error is used to estimate the precision of a function (mean) and to extrapolate information from a sample to a larger population. The Statistical Package for Social Sciences (version 94E) was used for all data.

## Discussion

The synthesis of ZnO NPs and Zn(cur)O NPs was conducted according to the methodology described by Moussawi et al. [28]. Before evaluating their antidepressant effects, a thorough examination of the physical and chemical properties of the nanoparticles was performed due to their significant impact on their physiological behavior [35]. The morphology and dimensions of the synthesized particles were examined using various techniques, including transmission electron microscopy (TEM), scanning electron microscopy (SEM), and dynamic light scattering (DLS). Analysis by SEM indicated that ZnO NPs exhibited a spherical shape, whereas Zn(cur)O NPs had a rod-like appearance. TEM images revealed that both ZnO NPs and Zn(cur)O NPs possessed hexagonal shapes, with average diameters of 23.6 ± 1.46 nm and 23.03 ± 5.2 nm, respectively. However, the average sizes determined by DLS were 220 ± 11.74 nm for ZnO NPs and 342 ± 22 nm for Zn(cur)O NPs. This discrepancy in size values between the two methods can be attributed to the fact that DLS measurements encompass the particle size and its solvation shell. In contrast, TEM provides size measurements of individual particles, excluding the solvation shell [36]. Particle stability was assessed using zeta potential (ZP), indicating particles’ tendency to aggregate or remain dispersed. The ZP values of ZnO NPs and Zn(cur)O NPs were determined to be − 19.6 ± 4.5 mV and − 25.6 ± 4.61 mV, respectively [37]. These results indicate low stability for ZnO NPs and high stability for Zn(cur)O NPs. The enhanced stability of Zn(cur)O NPs can be attributed to the strong interaction between curcumin and ZnO, as described by Moussawi et al. [38].

The blood–brain barrier (BBB) serves as a highly selective barrier that prevents the entry of external substances, including pharmaceuticals, into the brain [39]. However, nanoparticles offer a potential solution as drug delivery systems to overcome this obstacle. Upon oral absorption, ZnO nanostructures can either enter the brain through neural transport or breach the BBB. Among the proteins found on the surface of ZnO-NPs, apolipoprotein E plays a crucial role in facilitating the passage of nanoparticles through the BBB [40]. In this study, the antidepressant effects of curcumin, ZnO NPs, and Zn(cur)O NPs were evaluated using an animal model of depression induced by reserpine. Reserpine was chosen to cause depression as it reduces motor activity and depletes monoamines, which are the primary neurotransmitters involved in mood regulation [39]. Monoamine dysfunction and neuroinflammation are recognized as major contributors to depression, as reported by Yuanzhen et al. (2019) and Belmaker et al. (2008) [41, 42]. Additionally, disrupted motor activity has been associated with deficiencies in neurobiological processes [41, 42].

FST is a common test used to show the development of depression in animal models. The observed decrease in motor activity is indicated by increased immobility time and decreased time of struggling and swimming in the FST. Moreover, the OFT data revealed a decrease in crossed squares, rearing, and grooming following 15 days of reserpine injections daily. The time when the animals are immobilized and unable to get out of the water correlates with depressive-like actions [43].

The present decrease in 5-HT, NE, and DA is due to reserpine’s irreversible inhibitory impact on monoamine vesicular absorption. These findings are similar to the study of Ikram et al., who observed that acute treatment of Wistar rats with a low dose of reserpine-induced motor dysfunction and a substantial reduction in DA, NE, and 5-HT in the brain. Recent studies indicate that repeated reserpine administration may be utilized as a practical and cost-effective animal model for the progression of depression for antidepressant compound validation [44].

Curcumin (diferuloylmethane) has a wide range of pharmacological actions, such as monoamine replenishment [45], anti-inflammatory properties [46], and strong antioxidant function. Turmeric, a yellow curry spice, contains polyphenol curcumin, which has been us in traditional Indian dishes and herbal treatments for a long time [47]. In the FST, immobility reflects the desperate behavior of rats, and it is used as a sign of depression’s onset. Furthermore, a wide range of clinically effective antidepressant medicines may help to reduce immobility. This behavioral method is useful for identifying new antidepressants [48]. The current evidence clearly shows that curcumin reduces the immobility time and increases the serotonin level in the hippocampus and striatum. This might be due to the inhibitory result of curcumin on indoleamine 2,3-dioxygenase, the enzyme responsible for the degradation of the serotonin precursor, tryptophan, which increases serotonin. Curcumin’s antioxidant properties, communicated through MDA levels and enhanced catalase activity, may also play a role in its antidepressant efficacy, particularly as these effects are associated with increased motor activity [49].

ZnO NPs may improve behavioral and cognitive dysfunction in mice with depressive-like behavior via increasing synaptic plasticity in neurons and the area between the perforant pathway and the dentate gyrus [50]. In contrast, other studies showed that mice treated with ZnO NPs exhibited a modification of brain monoamines, ions, and histological impairment [51].

In the present study, after injecting rats with reserpine for 15 days, they were treated daily with curcumin, ZnO NPs, and Zn(cur)O NPs for 10 days, which increased the motor activity of animals. Raising and grooming increased this; however, only ZnO NPs raised rearing to the control level. Moreover, only curcumin and ZnO NPs increased grooming to control. Also, in the FST, struggling and swimming times increased significantly when rats were treated with curcumin and or Zn(cur)O NPs to that of the ZnO NPs exhibited values above the control. Using curcumin, Zn(cur)O NPs and ZnO NPs were more effective at decreasing immobility time.

Increased levels of reactive oxygen species (ROS) can lead to lipid peroxidation, forming malondialdehyde (MDA) and damage to cell membrane lipids. Clinical studies have shown that individuals with severe depression exhibit higher blood MDA levels than control groups [52]. Furthermore, research has indicated that MDA levels are elevated during depression and that antidepressant therapy can reduce lipid peroxidation levels [53, 54]. Glutathione (GSH), an endogenous antioxidant, is crucial in DNA repair and regulates various metabolic processes [55]. Individuals with depression have been found to have lower GSH levels [56, 57]. Catalase (CAT), an important antioxidant enzyme, converts hydrogen peroxide into oxygen and water. Clinical trials have demonstrated that catalase activity decreases when excessive oxidative stress leads to catalase deficiency, increasing levels of hydrogen peroxide and ROS in the body [58]. However, some studies have shown increased catalase activity during acute periods of depression [59–61].

According to the findings of this study, all three treatment formulations improved the oxidative stress status, as evidenced by changes in MDA, CAT, and GSH levels in the cortex, hippocampus, and striatum. Treatment with ZnO NPs and Zn(cur)O NPs significantly reduced MDA levels in the brain compared to the control group. Additionally, all treatment formulations significantly increased GSH levels compared to the control. In the striatum, all three treatments significantly decreased MDA levels compared to the animal model of depression. Notably, Zn(cur)O NPs led to a substantial increase in GSH levels and significantly increased CAT activity in the hippocampus compared to the control group.

Monoamine neurotransmitters play a crucial role in brain neurochemistry. Norepinephrine (NE) controls behavior, attention, prefrontal cortex activity, memory processing, and behavior [62, 63]. The original catecholamine theory of major depression suggests that depletion of NE at key synapses contributes to the development of depression through changes in the central nervous system [64]. Serotonin (5-HT) and its receptors have a modulatory effect on almost all brain functions, and dysregulation of the serotonergic system has been implicated in the pathogenesis of various psychological and neurological disorders [65, 66]. Dopamine (DA) is a neurotransmitter that regulates reward, motivation, working memory, and attention [67]. In the context of the interaction theory between depression and impaired DA transmission, compensatory upregulation of D2 receptor density in the basal ganglia/cerebellum has been observed in depressed patients compared to healthy individuals [68]. These findings highlight the significance of monoamine neurotransmitters in the etiology of depression and emphasize the role of NE, 5-HT, and DA in regulating various cognitive and emotional processes in the brain.

After three different therapies, there were significant improvements in monoamine levels in the cortex, hippocampus, and striatum. Among them, Zn(cur)O exhibited a promising effect by significantly increasing norepinephrine (NE) levels above the control group in the cortex. In the hippocampus, all three treatments effectively increased serotonin (5-HT) levels compared to the depression model. However, treatment with Zn(cur)O NPs demonstrated superior efficacy in elevating 5-HT levels in the control group. Similarly, Zn(cur)O NPs significantly increased 5-HT levels above the control values in the striatum. These findings indicate that Zn(cur)O has a more pronounced influence on restoring reserpine-induced depletion of monoamines compared to free curcumin or ZnO NP therapy. The selective impact of Zn(cur)O NPs can be attributed to several factors. Curcumin exerts its antidepressant properties by inhibiting monoamine oxidase activity, leading to elevated monoamine transmitters 5-HT and DA levels. The rise in 5-HT levels is associated with increased 5-HT receptor 1A mRNA expression. Additionally, curcumin exhibits anti-inflammatory properties by inhibiting enzymes involved in oxidative stress and apoptosis, including nuclear factor kappa-light-chain-enhancer of activated B cells (NF-kB), tumor necrosis factor-alpha (TNFα), and interleukin 6 (IL-6).

Furthermore, curcumin has demonstrated antidepressant effects by increasing the levels of serotonin (5-HT), dopamine (DA), and norepinephrine (NE) [69]. Moreover, utilizing ZnO nanoparticles (ZnO NPs) as a potential formulation for antidepressant purposes enhances curcumin’s consistency, efficacy, and delivery. Zinc (Zn) plays a crucial role in safeguarding the blood–brain barrier (BBB) against oxidative stress caused by free radicals. It is essential for synthesizing coenzymes involved in biogenic amine metabolism thereby maintaining brain homeostasis and preventing neurological disorders [70]. Zinc is known to regulate calcium reflux and prevent oxidative stress. Zinc deficiency can lead to the activation of nitric oxide synthase (NOS), mitochondrial dysfunction, and oxidative stress due to calcium influx [71]. Consequently, the antidepressant effects of ZnO NPs may be potentiated through their conjugation with curcumin, thereby offering a synergistic therapeutic approach.

## Conclusion

The data presented in this study demonstrates that the effectiveness of native curcumin in restoring oxidative stress and neurochemical changes associated with depression in the rat brain is limited. This ineffectiveness can be attributed to the low bioavailability of curcumin due to its poor water solubility. However, when curcumin is combined with ZnO nanoparticles (ZnO NPs), the pharmacokinetics of curcumin are enhanced, resulting in increased availability. This improved bioavailability may account for the remarkable antioxidant activity observed with Zn(cur)O NPs compared to curcumin and ZnO alone. Furthermore, the augmented bioavailability of curcumin through its conjugation with ZnO NPs enhances its inhibitory effects on monoamine oxidase, an enzyme responsible for the degradation of monoamines. This dual action leads to an elevation in monoamine levels and a reduction in the production of free radicals generated during monoamine oxidative catabolism.

Finally, the conjugation of curcumin with ZnO NPs offers a promising strategy to enhance its therapeutic efficacy by addressing the challenge of low bioavailability due to limited water solubility. This approach capitalizes on curcumin’s potent antioxidant and monoamine-modulating properties, thus holding potential for treating depression. The study findings provide valuable insights into the role of Zn(cur)O NPs as a novel therapeutic avenue for managing depressive disorders.

### Supplementary Information

Below is the link to the electronic supplementary material.

Supplementary file1 (DOCX 104 KB)

## Data Availability

The datasets generated during and/or analyzed during the current study are available from the corresponding author on reasonable request.
