# Supplementary material for: Evaluation of the Therapeutic Effect of Curcumin-Conjugated Zinc Oxide Nanoparticles on Reserpine-Induced Depression in Wistar Rats
Source: Biol Trace Elem Res. 2023 Sep 15;202(6):2630–44. doi: 10.1007/s12011-023-03849-z (PMC11052778; doi:10.1007/s12011-023-03849-z)
Supplement: Supplementary file 1 — Supplementary file1 (DOCX 104 KB) [file 12011_2023_3849_MOESM1_ESM.docx]

1. **Fourier-transform infrared (FTIR) spectroscopy discussion**


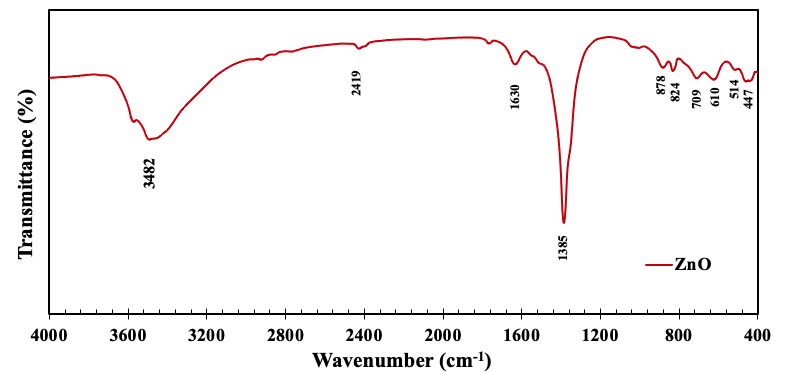


**Fig. S1**. FTIR analysis of ZnO

The analysis of the FTIR data has revealed a comprehensive spectrum characterized by distinct peaks at various wavenumbers, as shown in Fig. S1. The presence of peaks at 878 cm^-1^ and 824 cm^-1^ aligns remarkably well with the anticipated vibrational modes of zinc oxide (ZnO). These peaks correspond precisely to the vibrational modes associated with Zn-O bonds within the ZnO lattice structure. Additionally, the observation of peaks at 709 cm^-1^, 610 cm^-1^, and 514 cm^-1^ further reinforces the identification of ZnO. These specific wavenumbers have been consistently reported as characteristic lattice vibrations of ZnO in literature [1]. The appearance of peaks at 3482 cm^-1^ and 2419 cm^-1^, attributed to hydroxyl groups and atmospheric CO_2_ absorption, respectively, is not unexpected in IR spectra and does not negate the evidence for ZnO. Furthermore, the peaks at 1385 cm^-1^ and 1630 cm^-1^ are ascribed to primary and secondary alcohol in-plane bend or vibration, and their presence suggests potential contributions from organic compounds.

1. **UV-vis. spectroscopy discussion**

The UV spectroscopy analysis of the synthesized ZnO material revealed a distinct absorption band at 360 nm, as shown in Fig. S2, precisely matching the characteristic bandgap energy of ZnO reported in the literature. This correspondence strongly supports the successful synthesis of ZnO. The sharp and well-defined nature of the absorption peak further signifies the material's high crystalline quality. This observed absorption band aligns with well-established references [2, 3] for ZnO.


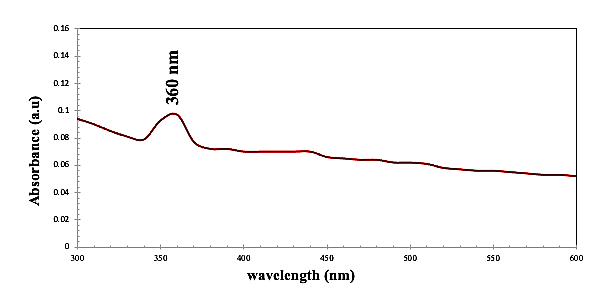


**Fig. S2**. UV-vis. spectroscopy analysis of ZnO

**References**

1. Jayarambabu N (2014) Germination and Growth Characteristics of Mungbean Seeds (Vigna radiata L.) affected by Synthesized Zinc Oxide Nanoparticles. International Journal of Current Engineering and Technology 4:5

2. Jang H, Kwon D-K, Kim DH, Myoung J-M (2022) Characteristics of flexible ZnO nanorod UV photodetectors processed by using a direct silicon etching transfer method. J Mater Chem C Mater 10:6805–6811. https://doi.org/10.1039/D2TC00377E

3. Alamdari S, Sasani Ghamsari M, Lee C, et al (2020) Preparation and Characterization of Zinc Oxide Nanoparticles Using Leaf Extract of Sambucus ebulus. Applied Sciences 10. https://doi.org/10.3390/app10103620
